# Increased Neuroplasticity in Frontal Cortex to Reduce Compulsive Behavior: A Preclinical tDCS Study in Male Rats

**DOI:** 10.1007/s12035-025-05218-4

**Published:** 2025-07-23

**Authors:** Manuela Olmedo-Córdoba, Angeles Prados-Pardo, Elena Martín-González, Margarita Moreno-Montoya

**Affiliations:** 1 Department of Psychology, Clinical and Experimental, Neuroscience Research Group CTS280 and CIBIS (Centro de Investigación Para El Bienestar y La Inclusión Social) Research Center, University of Almería, Ctra. Sacramento, S/N, 04120 Almería, Spain; 2 Área de Psicobiología, Facultat de Ciències de la Salut, Universitat Jaume I, Castellón de la Plana, Spain

**Keywords:** Executive function, Compulsive behavior, Schedule-induced polydipsia, Transcranial direct current stimulation, Serotonin 5-HT2A receptor, Gene expression, Neuroplasticity

## Abstract

Compulsive behavior is a potential transdiagnostic symptom highly present in different neuropsychiatric disorders, including obsessive–compulsive disorder (OCD), anxiety, schizophrenia, and addiction. Transcranial direct current stimulation (tDCS), a non-invasive neurostimulation technique, has been proposed as an effective and safe therapeutic strategy for reducing compulsive behavior. However, its underlying molecular mechanisms remain unclear. In the present study, we assessed whether anodal tDCS treatment reduces compulsivity through neuroplasticity mechanisms in male Wistar rats selected by high compulsive drinking on schedule-induced polydipsia (SIP). Compulsive rats received low-intensity direct current stimulation (0.5 mA) over the frontal cortex (FC) once a day for 8 consecutive days for 20 min, compared to a sham group without stimulation. tDCS treatment did not induce a significant reduction in compulsivity on SIP. However, RT-qPCR analyses revealed that tDCS led to a significant increase in different neuroplasticity markers, such as *Htr2a*, *Grin1*, *Bdnf*, *Ngf*, and *Scn2a* in the FC of compulsive rats compared to sham treatment. In contrast, tDCS treatment did not induce any change in the neuroplasticity markers in the amygdala. These data suggest that tDCS might be able to induce neuromodulation in the FC by an increase in neuroplasticity gene expression, despite not observing significant differences in compulsive behavior on SIP. Our findings also suggest that future studies employing neuromodulation techniques should aim to target neuroplastic changes within the amygdala, with the potential to reduce compulsive behaviors.

## Introduction

Compulsivity is a transdiagnostic trait present in different neuropsychopathological disorders such as obsessive–compulsive disorder (OCD), anxiety, schizophrenia, autism, gambling, and substance use disorder, among others [1–3]. Compulsive behavior is described as a repetitive, persistent, inflexible, and maladaptive act characterized by its disconnection from environmental contingencies and lack of relevance to the final goal [4]. One of the main psychopharmacological treatments has been selective serotonin reuptake inhibitors (SSRIs) [5, 6], which act through the serotonin 5-HT2A receptor and glutamatergic signaling in the prefrontal cortex [7, 8]. However, most OCD patients have incomplete or no benefit from this treatment [6, 9, 10], highlighting the need for alternative therapeutic strategies.

In the last two decades, transcranial direct current stimulation (tDCS) has emerged as a promising non-invasive neuromodulation technique for neuropsychopathological disorders, such as OCD [11–16], anxiety [17–20], addiction [21–24], depression [25–27], and ADHD [28, 29]. tDCS treatment modulates neuronal excitability by the application of a low-intensity electrical current through the scalp electrodes [30–34], with anodal stimulation increasing cortical excitability and cathodal stimulation decreasing [30, 31]. The underlying mechanism of this modulation seems to implicate serotonergic, glutamatergic, and other neuroplasticity biomarkers. Thus, the action of tDCS on the serotonin system [35] might explain its ability to enhance SSRIs’ efficacy in depression and OCD [36–39]. Moreover, tDCS as a single treatment increased glutamate/glutamine (Glx) levels at electrode sites [40–44]; therefore, some studies reported that it enhanced NMDA receptor modulators treatment [45, 46]. Finally, tDCS in preclinical studies increased brain-derived neurotrophic factor (BDNF) [47]. However, the neuromolecular mechanisms underlying the therapeutic effects of tDCS on compulsivity remain poorly understood and might be addressed through preclinical models.

One relevant preclinical model for the study of compulsivity is schedule-induced polydipsia (SIP), in which food-deprived animals develop a compulsive adjunctive drinking behavior under an intermittent food reinforcement [48–50]. High drinker rats selected by SIP have been characterized as a compulsive phenotype by its behavioral and neurobiological alterations [49]. Compulsive rats on SIP have shown (1) behavioral inflexibility [51–54], cognitive and motor impulsivity [51, 55], and socioemotional alterations [54, 56]; (2) altered corticolimbic circuitry, by a reduced volume of medial prefrontal cortex (mPFC) and an increased basolateral amygdala [57, 58]; and (3) at the molecular level, decreased 5-HT2A receptor binding in the frontal cortex (FC) and the amygdala [59], *Htr2a*, *Grin1*, and *Bdnf* mRNA levels in the FC [60] and *Arc* in the locus coeruleus [61].

 Based on the previous evidence, the present study hypothesized that anodal tDCS stimulation might restore molecular corticolimbic functions in compulsive rats selected by SIP, exploring the underlying mechanisms of its potential therapeutic role. To achieve this goal, anodal tDCS was applied to the scalp in the FC region of compulsive male rats selected by SIP. Then, we assessed its effects on compulsive behavior in SIP and on genetic expression linked to neuroplasticity biomarkers in the FC and the amygdala. These brain areas were selected according to our previous data, in which the molecular differences of compulsive rats were expressed [59–61]. The neuroplasticity assessment included serotonergic genes (*Htr2a* and *Htr2c*), glutamatergic genes (*Grin1* and *Grin2a*), and other neuroplasticity markers such as *Bdnf*, nerve growth factor (*Ngf*), early growth response 1 (*Egr1*), activity-regulated cytoskeleton-associated protein (*Arc*), and sodium voltage-gated channel alpha subunit 2 (*Scn2a*). The identification of the tDCS-induced neuroplastic changes will help to enhance new treatments for compulsivity-related disorders, highlighting tDCS as a therapeutic alternative alone or in combination with pharmacological strategies to target these molecular mechanisms.

## Materials and Methods

### Animals

A total of 30 male Wistar rats from Envigo (Barcelona, Spain), weighing between 250 and 275 g, were used for the current experiment. The animals were housed in home boxes four by four (50 × 35 × 20 cm) with standard conditions: a temperature of 21 ± 2 °C and light/dark cycles of 12 h each (the dark cycle began at 08:00 h). From their arrival, they received an amount of water and food “ad libitum” (unlimited) and environmental enrichment (wooden blocks). After 5 days, the animals were gently handled for a week. Before starting the behavioral tests, the animals were subjected to gradual deprivation until reaching 85% of their free weight, a weight that was maintained during the entire experiment. All experiments comply with the ARRIVE guidelines and have been carried out in accordance with the EU Directive 2010/63/EU for animal experiments and the National Spanish Research Royal Decree 53/2013 for the Care and Use of Laboratory Animals. The authors declare that the research shows commitment to the 3Rs principle (replacement, reduction, refinement).

### Experimental Design

**Fig. 1:**
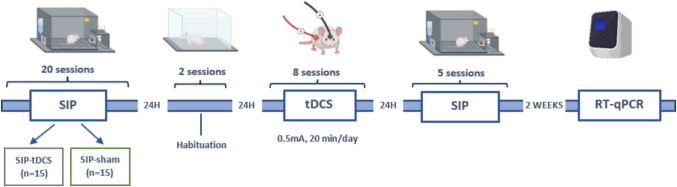
The experimental procedures are illustrated in a timetable. SIP, 20 sessions: after schedule-induced polydipsia acquisition, rats were equally distributed to either the sham (SIP-sham, *n* = 15) or transcranial direct current stimulation tDCS group (SIP-tDCS, *n* = 15). Habituation, 2 sessions: animals were habituated to the chamber prior to the tDCS treatment. tDCS, 8 sessions: rats received sham or tDCS treatment according to the experimental group. SIP, 5 sessions: re-assessment of compulsive behavior on SIP after sham or tDCS treatment. RT-qPCR: animals were euthanized 2 weeks after the last SIP session and brain samples were extracted to analyze gene expression by reverse transcription quantitative polymerase chain reaction. This figure is created by BioRender.com

The order of the experimental events is summarized in Fig. 1.

Male Wistar rats were exposed to 20 sessions (one session/day) of SIP acquisition, before tDCS treatment, in eight operant chambers (MED Associates, USA). SIP procedure and apparatus have been previously described [59]. Before SIP acquisition and over two consecutive days, the amount of water consumed by each rat in 60 min was measured as baseline. There was unlimited access to a bottle of water, and 60 food reward pellets were placed together (Noyes 45-mg dustless reward pellets; PHYMEP, France). SIP: The animals were then exposed to a fixed-time 60-s (FT-60 s) schedule of food pellet presentation in 60 min sessions, and water consumption was measured by weighing the bottle before and after each session. After 20 SIP sessions, rats showed compulsive drinking behavior above < 12 ml according to Íbias et al. [62].

Once compulsive behavior on SIP was established, the total population of rats (*N* = 30) was equally distributed into two equivalent experimental groups, according to either the sham (SIP-sham, *n* = 15) or tDCS treatment condition (SIP-tDCS, *n* = 15). After eight sessions of tDCS treatment (one session/day), both groups were re-assessed on SIP during five sessions for compulsive drinking assessment. Fifteen days after the last session of SIP, animals were euthanized by rapid decapitation under anesthesia (4% isoflurane inhalation); this time has been used previously as a washout period to avoid any possible effects following compulsive water intake on SIP [59]. Brain tissue samples of the FC and the amygdala were extracted and immediately frozen on dry ice to prevent degradation of the RNA. The samples were then stored at − 80 °C until gene expression analyses by reverse transcription quantitative polymerase chain reaction. RT‑qPCR was then performed to assess the effects of tDCS on gene expression of different neuroplasticity markers in compulsive rats.

### Stimulation Protocol by tDCS

Anodal tDCS was applied using a non-invasive and ecological method in freely moving animals in a chamber. During the stimulation, the animals were neither immobilized nor anesthetized but were able to move freely within the chamber, enhancing the translational relevance of the procedure. To minimize stress associated with exposure to a novel environment, animals were habituated to the chamber during two separate sessions conducted on different days. Anodal stimulation consisted of a low-intensity direct current (0.5 mA) applied for eight consecutive sessions (once a day for 20 min). The current source was a 355-WPI stimulator (World Precision Instruments). This protocol was selected according to previous studies [33, 63–65]. To avoid an abrupt start or end of the current, the intensity was modulated at an interval of 20 s until the maximum (0.5 mA) and minimum (0 mA) points were reached [66]. Electrodes with adhesive gel were trimmed to 1.5 cm^2^. For correct adherence, the animal’s head was shaved (under isoflurane 4%) and fixed with transparent tape (OMNIFILM) to prevent removal. The electrodes were attached to the scalp to emulate the tDCS method applied in clinical studies [33, 65]. Specifically, the anode was placed at the midpoint between the lateral angles of both eyes (supraorbital area) and the cathode on the ventral torso [64]. The animals with sham stimulation had the same electrode placement and fixation; however, during the procedure, the stimulator remained in the “off” position [67]. According to the stimulation parameters (intensity and electrode size), the current density used was 3.33 A/m^2^ as it has been reported in studies that a current density higher than 142.9 A/m^2^ is associated with brain damage [68].

### Gene Expression: Reverse Transcription Quantitative Polymerase Chain Reaction

RNA samples were extracted from the FC and the amygdala of a total of 14 animals randomly selected from each group (*n* = 7 SIP-sham, *n* = 7 SIP-tDCS). The RNA was purified using Trizol reagent (Invitrogen) following the manufacturer’s instructions. The RNA was quantified using a fluorescence signal with Qubit® fluorometer (Fisher Scientific). The TURBO ® DNAse-I kit (Ambion) was used to remove genomic DNA (gDNA) contamination. Then, contamination-free total RNA was retrotranscribed into complementary DNA (cDNA) using Maxima First Strand cDNA Synthesis Kit® (Thermo Scientific). Gene expression analysis was carried out by RT-qPCR assays in a Step-One Real-Time PCR System (Applied Biosystems). All reactions contained cDNA, the pair of primers (see Table 1), the nuclease-free water, and the SYBR Green Master Mix (Fisher Scientific). For each primer, adequate efficiency was measured by serial dilutions (1:10). Primers designed in exon sections of the Gapdh gene were used as a housekeeping gene, while intron primers checked for gDNA contamination. All data were obtained and analyzed using StepOne real-time PCR Systems software (v2.2.2, Applied Biosystems). For a more detailed protocol, see Perez-Fernandez et al. [69].

**Table 1:** Primers selected for the RT-qPCR study. Described from left to right: gene ID (*Rattus norvegicus*), forward primer, reverse primer, and source. GAPDH, glyceraldehyde 3-phosphate dehydrogenase. *Htr2a* and *Htr2c*, serotoninergic receptor 2a and b. *Grin1* and *Grin2a*, glutamate ionotropic receptor NMDA type subunit 1 and 2a. *Bdnf*, brain-derived neurotrophic factor. *Ngf*, nerve growth factor. *Egr1*, early growth response 1. *Arc*, activity-regulated cytoskeleton-associated protein. *Scn2a*, sodium voltage-gated channel alpha subunit 2

Gene ID (Rattus)	Forward primer	Reverse primer	Source
Gapdh (Intron)	ctgggtggctcaaggaata	ctgggtggctcaaggaata	[70]
Gapdh (Exon)	cttcaccaccatggagaag	catggactgtggtcatgag	[70]
*Htr2a*	atgctgctgggtttccttgt	atcgcacagagcttgctagg	Own design
*Htr2c*	ttggactgagggacgaaagc	ggatgaagaatgccacgaagg	[71]
*Grin2a*	agttcacctatgacctctacc	gttgatagaccacttcacct	[72]
*Grin1*	atggcttctgcatagacc	gttgtttacccgctcctg	[72]
*Bdnf*	ggtcacagcggcagataa	ccgaacatacgattgggtag	Own design
*Ngf*	acgcagctttctatcctggc	ctgcctgtacgccgatcaaa	Own design
*Egr1*	aacaaccctacgagcacctg	accagcgccttctcgttatt	Own design
*Arc*	tctcagggtgagctgaagca	ctggtatgaatcactgctgggg	Own design
*Scn2a*	tgcactggagactgctacat	ctcgcgtaagaaagtgctga	Own design

### Statistical Analysis

SIP acquisition pre-tDCS treatment data were analyzed using a one-way repeated-measures analysis of variance (ANOVA), with “sessions” (20 sessions) as the within-subject factor. The equal distribution of experimental groups was analyzed using a two-way repeated-measures analysis of variance (ANOVA), with “group” (SIP-sham and SIP-tDCS) as a between-subject factor and “sessions” (20 sessions) as the within-subject factor. For post-tDCS SIP, we calculated the percentage change in water intake using the following equation: [(water intake SIP post-tDCS/water intake SIP pre-tDCS) × 100] and the area under the curve (AUC), using the trapezoid method, of the daily percentage change in water intake for each subject over five consecutive days. Differences between SIP-sham and SIP-tDCS sessions were analyzed using Student’s t-tests (*T*-test). The differences in gene expression between SIP-sham and SIP-tDCS for each of the genes in the FC and the amygdala were analyzed using Student’s t-tests (*T*-test). When there was a violation of equality of variances, it was analyzed using the non-parametric Mann–Whitney *U* test. The data were assessed for equality of variances using Levene’s test. Post hoc analyses were performed with Bonferroni test corrections. Statistical significance was established at *p* < 0.05. Partial eta-squared values of 0.01, 0.06, and 0.14 and Cohen’s *d* values of 0.2, 0.5, and 0.8 are considered to reflect small, medium, and large effects, respectively [73]. All analyses were performed with Statistica® software (version 8.0), and all figures were made using GraphPad Prism 9.

## Results

### Behavioral Assessment: SIP Acquisition Pre-tDCS and Post-tDCS Treatment

**Fig. 2:**
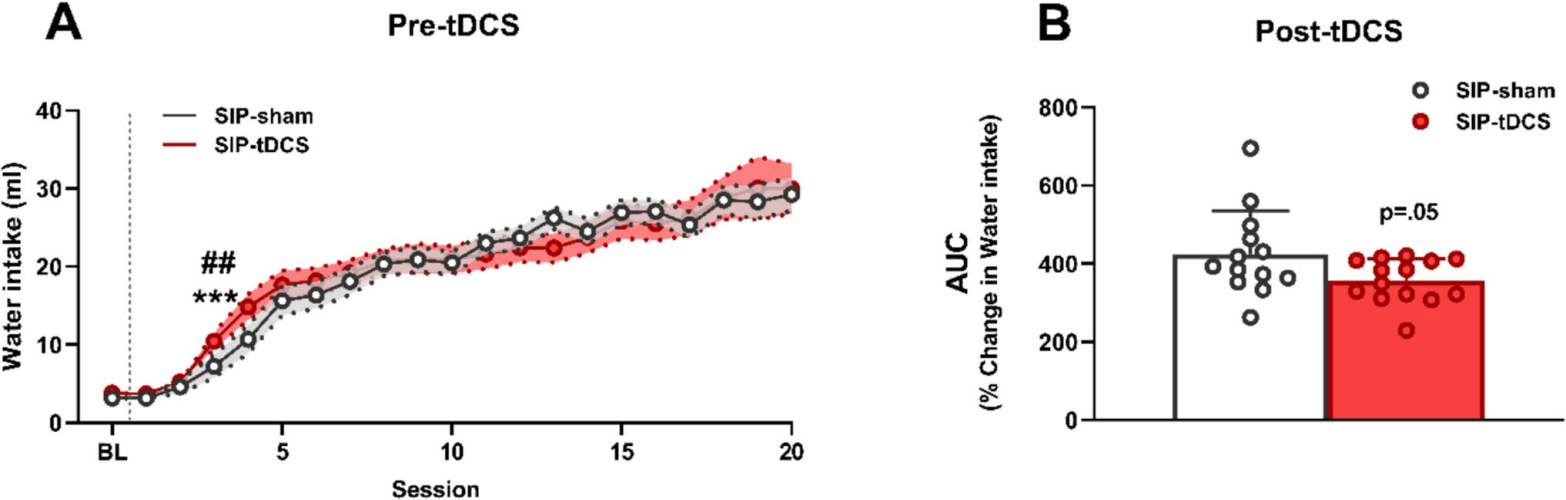
SIP water intake. Pre-tDCS acquisition is shown by the mean (± SEM) of water intake in FT-60 across 20 sessions (**A**), SIP-sham (*n* = 15) and SIP-tDCS (*n* = 15). The statistical analysis indicates significant differences from session 3 onwards compared to session 1 for the SIP-sham group (## *p* < 0.01) and for the SIP-tDCS group (**p < 0.001). SIP post-tDCS (**B**), shown by the mean AUC of the percentage change in water intake over 5 days (± SEM) in SIP-sham (*n* = 13) and SIP-tDCS (*n* = 14) rats (*p* = 0.05)

Figure 2A shows SIP acquisition through 20 sessions, by means of water intake in the total population of rats (*N* = 30), according to the SIP-sham (*n* = 15) and SIP-tDCS (*n* = 15) groups before tDCS treatment. SIP acquisition was evidenced in the water intake in both groups of rats (*F* (19,551) = 74.03, *p* < 0.001, *η*2*p* = 0.72) by repeated-measures ANOVA session effect. Post hoc analysis revealed that there was a significant increase in water intake in session 3 (*p* < 0.01; *d* = 1.32) compared to session 1. The analysis of SIP acquisition in the two experimental groups showed similar patterns of compulsive drinking with a mean water intake of 27.4 ± 1.9 ml for SIP-sham and 26.8 ± 2.0 ml for SIP-tDCS during the last 5 days of SIP (Fig. 2A). The equal distribution of experimental groups on SIP is also demonstrated through the analysis of water consumption, where repeated-measures ANOVA did not reveal significant differences according to the interaction between SIP acquisition sessions and group effect (*F* (19, 532) = 1.01, *p* > 0.05). Instead, SIP acquisition was significant in water intake due to session effect (*F* (19, 532) = 74.07, *p* < 0.001, *η*2*p* = 0.72). Moreover, both groups showed SIP acquisition by a significant water intake from session 3 onwards: post hoc analysis in the SIP-sham group (*p* < 0.01; *d* = 1.74) and the SIP-tDCS group (*p* < 0.001; *d* = 1.56) at session 3 compared to session 1. Figure 2B illustrates the effects of anodal tDCS treatment in compulsive water intake on SIP. Anodal SIP-tDCS rats showed a non-significant reduction in the percentage of change in water intake AUC (357.36 ± 56.19 vs. 425.32 ± 110.65) compared to SIP-sham rats (*t* = − 2.03, *df* = 25, p = 0.05; *d* = 0.78).

### Gene Expression

#### Relative Expression of Serotonergic Genes

**Fig. 3:**
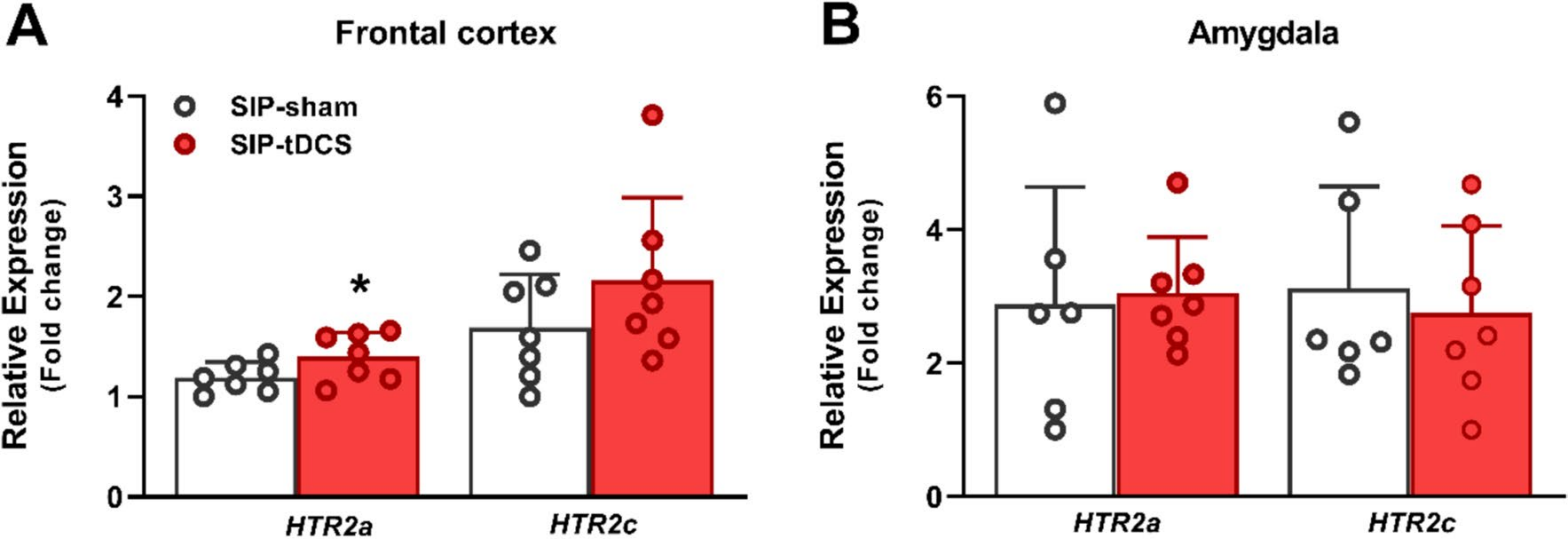
Serotoninergic markers. Relative expression (± SEM) of *Htr2a* and *Htr2c* of SIP-sham (*n* = 6 or 7) and SIP-tDCS rats (*n* = 7) in the frontal cortex (**A**) and the amygdala (**B**). Statistical analyses indicate significant differences between SIP-sham and SIP-tDCS rats (*p < 0.05) in the relative expression of *Htr2a* in the frontal cortex

The effects of anodal tDCS treatment in *Htr2a* and *Htr2c* mRNA expression levels in the FC and the amygdala are shown in Fig. 3. In the FC (Fig. 3A), SIP-tDCS rats exhibited an increased *Htr2a* mRNA expression levels (*t* = − 1.95, df = 12, *p* = 0.038; *d* = 1.04) compared to SIP-sham rats. However, tDCS had no significant effect on *Htr2c* mRNA expression levels (*t* = − 1.28, df = 12, *p* = 0.11). In the amygdala (Fig. 3B), tDCS treatment did not show any significant differences in *Htr2a* mRNA expression levels (*t* = − 0.23, df = 11, *p* = 0.59) or *Htr2c* (*t* = 0.47, df = 11, *p* = 0.32) between groups.

#### Relative Expression of Glutamatergic Genes

**Fig. 4:**
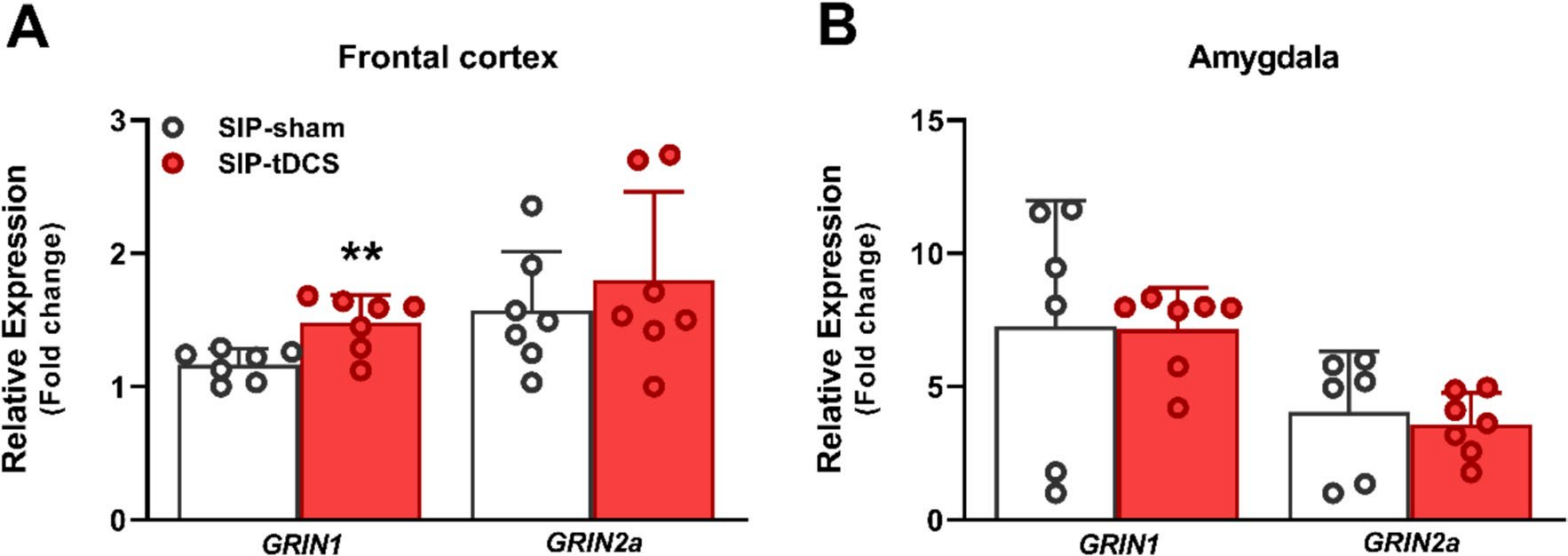
Glutamatergic markers. Relative expression (± SEM) of *Grin1* and *Grin2a* of SIP-sham (*n* = 6 or 7) and SIP-tDCS rats (*n* = 7) in the frontal cortex (**A**) and the amygdala (**B**). Statistical analyses indicate significant differences between SIP-sham and SIP-tDCS rats (**p < 0.01) in the relative expression of *Grin1* in the frontal cortex

Figure 4 shows the effects of anodal tDCS treatment on *Grin1* and *Grin2a* mRNA expression levels in the FC and the amygdala. In the FC (Fig. 4A), tDCS treatment increased *Grin1* mRNA expression levels in the SIP-tDCS group (*t* = − 3.50, df = 12, *p* = 0.002; *d* = 1.87), compared to the SIP-sham group. However, no differences were found in the modulation of *Grin2a* mRNA expression levels (*t* = − 0.75, df = 12, *p* = 0.23) between SIP-tDCS and SIP-sham rats. In the amygdala (Fig. 4B), anodal tDCS did not show any significant differences in *Grin1* mRNA expression levels (*t* = 0.05, df = 11, *p* = 0.48) or *Grin2a* (t = 0.47, df = 11, p = 0.32) compared to SIP-sham rats.

#### Relative Expression of Genes Related to Neuroplasticity Markers

**Fig. 5:**
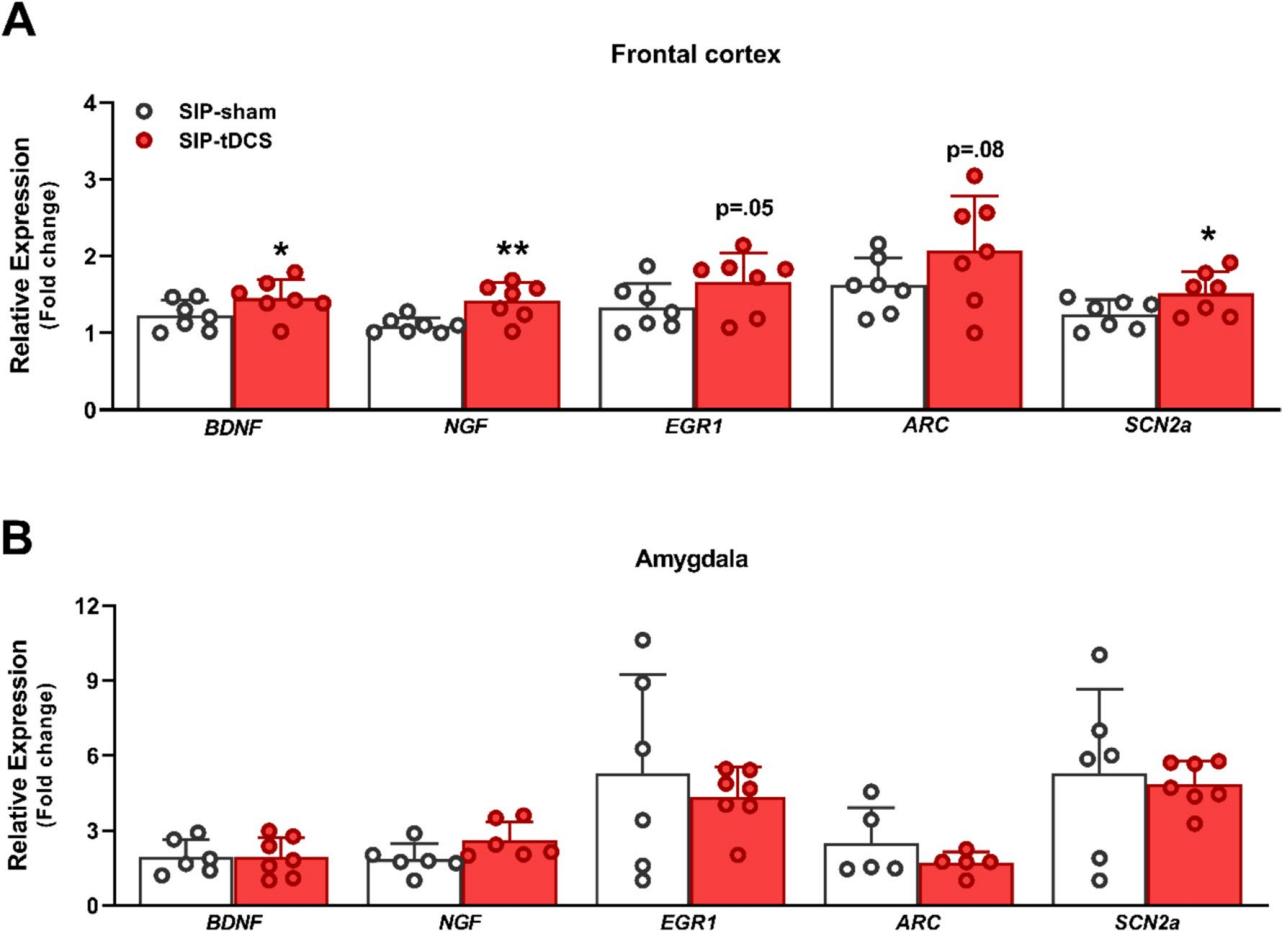
Neuroplasticity markers. The relative expression (± SEM) of *Bdnf*, *Ngf*, *Egr1*, *Arc*, and *Scn2a* of SIP-sham (*n* = 6 or 7) and SIP-tDCS rats (*n* = 7) in the frontal cortex (**A**) and the amygdala (**B**). Statistical analyses indicate significant differences between SIP-sham and SIP-tDCS rats (*p < 0.05; **p < 0.01) in the relative expression of *Bdnf*, *Scn2a* (*p < 0.05), and *Ngf* (**p < 0.01) in the frontal cortex

Figure 5 shows the effects of anodal tDCS treatment on the mRNA expression levels of other markers involved in neuroplasticity in the FC and the amygdala. Regarding the FC (Fig. 5A), SIP-tDCS rats showed significantly increased *Bdnf* (*t* = − 1.90, df = 12, *p* = 0.04, *d* = 1.02), *Ngf* (*t* = − 3.34, df = 12, *p* = 0.003, *d* = 1.79), and *Scn2a* mRNA expression levels (*t* = − 2.14, df = 12, *p* = 0.03, *d* = 1.14) compared to SIP-sham rats. Additionally, there was a non-significant trend towards increased *Arc* (*t* = − 1.52, df = 12, *p* = 0.08) and *Egr1* mRNA expression levels (*t* = − 1.73, df = 12, *p* = 0.05) between SIP-tDCS and SIP-sham rats. In the amygdala (Fig. 5B), the SIP-tDCS rat group did not exhibit any significant changes in *Bdnf* (*t* = 0.05, df = 11, *p* = 0.50), *Ngf* (*t* = − 1.95, df = 11, *p* = 0.16), *Scn2a* (*t* = 0.34, df = 11, *p* = 0.37), *Arc* (*t* = 1.19, df = 8, *p* = 1.13), or *Egr1* mRNA expression levels (Mann–Whitney *U* test, *p* = 0.47) compared to the SIP-sham rat group.

## Discussion

The present study investigated the behavioral and neuroplasticity effects of anodal tDCS neurostimulation in a preclinical model of compulsivity in male rats. The exposure to anodal tDCS neurostimulation in the FC (8 sessions, 20 min/day) in male rats did not induce a significant reduction in compulsive drinking on SIP compared to sham treatment. However, despite the lack of behavioral differences, tDCS induced a significant increase in gene expression level of serotoninergic, glutamatergic, and other neuroplasticity biomarkers such as *Htr2a*, *Grin1*, *Bdnf*, *Ngf*, and *Scn2a* in the FC of compulsive rats compared to sham treatment. In contrast, no significant differences were observed in gene expression levels in the amygdala. This is the first study that addresses the effectiveness of anodal tDCS in a preclinical model of compulsive behavior trying to evidence its neuroplasticity underlying mechanisms.

### The Effect of Anodal tDCS on Compulsive Behavior

Anodal tDCS over the FC did not induce a significant reduction in compulsive drinking on SIP. However, previous preclinical studies using similar stimulation parameters have demonstrated significant behavioral effects in other paradigms such as anxiety and fear extinction. Thus, a 7-day protocol (20 min/day) of anodal tDCS in the FC effectively reduced anxiety in the elevated-plus maze (EPM) in male Sprague–Dawley rats exposed to acute stress [74], and a 5-day protocol (two daily 20 min/session) in the FC improved fear extinction in mice [75]. In contrast, 1-day treatment in FC (1 day, 20 min) did not improve fear memory extinction [76]. These results suggest that the proven tDCS efficacy might also depend on the protocol of stimulation duration and frequency and could also rely on the behavioral task used for its assessment. In this sense, according to our study, the same stimulation tDCS protocol that successfully modulated anxiety and fear extinction is insufficient to significantly reduce compulsive behavior on SIP.

Clinical studies have highlighted the involvement of the cortico-striato-thalamo-cortical circuits in the pathophysiology of compulsivity in patients with OCD [77]. In particular, regions such as the orbitofrontal cortex (OFC), dorsolateral prefrontal cortex (dlPFC), anterior cingulate cortex (ACC), supplementary motor area (SMA), and limbic structures—including the amygdala and hippocampus—have been implicated in compulsive behavior [1, 77]. tDCS treatment has been shown to effectively reduce compulsive symptoms, particularly in treatment-resistant cases of OCD, by targeting the dlPFC, the OFC, and the cerebellum using both unilateral and bimodal montages. Cathodal stimulation over the left OFC, usually combined with anodal placement over the cerebellum or occipital region, has acutely reduced OCD symptoms measured by the Yale-Brown Obsessive–Compulsive Scale (Y-BOCS) [14, 78–80]. Similarly, tDCS had a greater efficacy using bimodal stimulation targeting both the OFC and the SMA [12]. However, despite the acute effects, some of these studies recognize a lack of an effective long-lasting reduction in compulsivity symptoms in patients with treatment-resistant OCD [78]. In this sense, review studies on tDCS and OCD have collated inconsistent findings across studies, pointing towards different causes such as methodological heterogeneity (e.g., electrode placement, polarity, number of sessions), limited sample size, and lack of sham-controlled designs [81]. Moreover, it is important to note that clinical tDCS studies have overlooked the relevant role of the amygdala in OCD [1, 77], possibly due to the cortical application of the technique. Therefore, the lack of a significant reduction in compulsive drinking on SIP might be related to the stimulation protocol parameters (intensity, duration, exposure), but also a possible individual genetic variability; this issue is further addressed in the following subsection. Based on clinical evidence, future preclinical studies should aim to optimize stimulation protocols and further investigate the potential of bimodal tDCS to enhance therapeutic efficacy.

### Anodal tDCS Effects on Genetic Expression in Compulsive Rats

All gene expression changes observed in this study were restricted to the FC, with no significant alterations detected in the amygdala. These findings suggest that the neuromodulatory effects of anodal tDCS in the present study are possibly confined to the stimulated cortical area, without extending to subcortical areas, in this case, the amygdala.

#### Serotonergic Genetic Expression

Anodal tDCS treatment in compulsive rats increased Ht*r2a* mRNA expression levels in the FC compared to untreated compulsive rats. However, no differences were found in *Htr2a* mRNA expression levels in the amygdala, nor in the *Htr2c* expression levels in both the FC and the amygdala. Extensive research carried out in our laboratory has characterized the serotonin 5-HT2A receptor as a hallmark of the compulsive phenotype in rats selected by SIP. Compulsive drinker rats on SIP have shown a reduced binding and mRNA expression levels of the 5-HT2A receptor in the FC compared to non-compulsive rats [59, 60]. Moreover, in neuropharmacological studies, the serotonin 5-HT2A/C receptor agonist DOI reduced compulsive drinking on SIP [59, 82]. These results are also in accordance with the reduced availability and lower binding of the serotonin 5-HT2A receptor in the FC and the efficacy of SSRI in OCD patients [76, 83]. On the other hand, the lack of effect of tDCS treatment in the gene expression of the 5-HT2C receptor might also be explained by the differences observed in neuropsychopharmacological studies on SIP [84, 85]. Moreover, a meta-analysis did not find significant associations between the HTR2C polymorphism and OCD [86]. Therefore, anodal tDCS might induce a therapeutic effect on compulsivity through the genetic expression changes in the 5-HT2A receptor. According to our results, previous preclinical and clinical studies have shown that neuromodulation treatment is able to induce changes in the serotonergic system. Thus, anodal and cathodal tDCS on the prefrontal cortex (PFC) (for 8 days, 10 × 2 min/day) increased concentrations of serotonin and its metabolite 5-HIAA in the FC and the dorsal striatum in rats [87]. Moreover, clinical studies have shown that SSRI are able to intensify the excitatory effects of anodal tDCS treatment [36, 37]. However, the differences in genetic sensitivity, due to variants of the serotonin transporter (5-HTTLPR), seem to have a role in the effects of tDCS on the serotoninergic system. Clinical studies have revealed that carriers of the short allele of the 5-HTTLPR polymorphism are characterized by amygdala hyperactivity, a condition that might reduce tDCS efficacy by interfering with cortical top-down regulation [35]. The hyperactivity of the amygdala in compulsive rats on SIP [88] may offer a potential explanation for the lack of significant tDCS effect on compulsive drinking. Future studies should further investigate individual differences in the amygdala hyperactivity and their interaction with the therapeutic effects of tDCS in reducing compulsive behaviors.

#### Glutamatergic Genetic Expression

Compulsive rats treated with anodal tDCS increased *Grin1* mRNA expression levels in the FC compared to untreated compulsive rats. However, no differences were found in *Grin1* mRNA expression levels in the amygdala, nor in the *Grin2a* gene in both the FC and the amygdala. This is the first study, to date, that investigates the effects of tDCS stimulation on the gene expression of glutamatergic markers such as Grin1 and Grin2a using preclinical models. A recent study in our laboratory revealed that rats with a compulsive phenotype exhibited a significant decrease in *Grin1* mRNA expression levels in the FC compared to the non-compulsive group of rats [60].

Previous preclinical studies on tDCS have shown modulation of the glutamate–glutamine cycle that might contribute to changes in glutamatergic receptor expression, including Grin1. Anodal tDCS stimulation in rats (for 7 days, 0.5 mA 30 min/day), applied with a disc electrode placed on the head, regulated glutamate release and reuptake by reducing the overexpression of NMDAR2A and AMPAR1 receptors and increasing the expression of excitatory amino acid transporters (EAAT2 and EAAT3) in the hippocampus [89]. A single session of bimodal tDCS in rats (1 day, 20 min), cathode on the forehead and anode in the hippocampus, increased glutamate uptake and glutamine synthesis activity via glutamine synthetase in hippocampal astrocytes [90]. However, findings from clinical studies on tDCS treatment have shown some inconsistencies regarding the neurometabolite Glx, measured by magnetic resonance spectroscopy. Some studies have reported increased Glx concentration under the anode in brain areas such as the parietal lobe [42], the PFC [40], and the occipital temporal region [41]. In contrast, other studies have found no significant changes in Glx levels in the dlPFC [91], while some have even reported decreased Glx concentrations in this region [43, 44].

#### Neuroplastic Markers Genetic Expression

Anodal tDCS treatment in compulsive rats increased *Bdnf*, *Ngf*, and *Scn2a* mRNA expression levels in the FC, with an observed trend to increase in *Arc* and *Egr1* expression levels, compared to untreated compulsive rats. No differences were observed in the neuroplastic markers in the amygdala. BDNF, NGF, ARC, and EGR1 are key genes involved in synaptic plasticity. BDNF promotes dendritic spine formation, synaptogenesis, and long-term potentiation (LTP) [92], while NGF regulates neuronal growth [93]. EGR1 (also known as Zif268) functions as an immediate early gene that is rapidly induced by synaptic activity and is crucial for LTP stabilization [94, 95]. Moreover, EGR1 upregulates ARC, a gene that directly modulates synaptic plasticity after neuronal activation [96, 97]. Preclinical studies on compulsivity have revealed reductions in BDNF, ARC, and EGR1 neuroplastic markers. In the SIP model, compulsive high drinker rats showed decreased *Bdnf* mRNA expression levels in the FC [60] and reduced *Arc* mRNA expression levels in the locus coeruleus [61]. Similarly, the induction of compulsive behavior in rats by quinpirole administration has been associated with reduced *Bdnf* mRNA expression levels in the OFC [98], decreased BDNF protein levels in serum [99], and reduced *Arc* gene expression in the hippocampus, but not in the mPFC [100]. In another preclinical study, adolescent rats displaying persistent reward-seeking despite foot shock punishment exhibited reduced *Egr1* mRNA expression in the anterior insular cortex [101]. Clinical studies have also reported reduced BDNF levels in both serum and plasma in patients with OCD [102, 103]. According to our findings, previous preclinical studies suggest that tDCS treatment may modulate these neuroplasticity markers. In rodents, anodal tDCS applied over the FC (for 8 days, 20 min/day) increased BDNF protein levels in the PFC [104]. Similarly, anodal stimulation on the FC in mice (for 5 days, 2 × 20 min/day) increased basal expression of Zif268 in both the FC and the striatum [105]. A more recent study in rats demonstrated that anodal tDCS on the parietal cortex in rats (targeting the M1 motor area; 7 days, 20 min) increased plasma BDNF levels [106]. Furthermore, the combination of bimodal tDCS (anodal and cathodal electrodes placed on the head using external anatomical landmarks; for 8 days, 20 min/day) and physical exercise elevated serum NGF levels in rats [107]. However, the same stimulation protocol failed to induce changes in NGF levels in the striatum and cerebellum [65].

SCN2A contributes to dendritic excitability in mature neocortical pyramidal cells, where its loss negatively affects excitatory synaptic function and neuronal plasticity [108]. In preclinical studies, animal models of Scn2a haploinsufficiency showed increased compulsivity-like behaviors: increased repetitive behavior in the marble burying test [109], increased fear conditioning and impaired extinction in the conditioned place preference test [110], and increased anxiety behavior in the open field test [111]. To date, no studies have examined the effects of tDCS on SCN2A expression. In our study, anodal tDCS increased the mRNA expression levels of *Scn2a* in the FC.

In summary, our findings suggest that anodal tDCS acts through multiple molecular mechanisms. Therefore, we observed an increased expression level of the *Htr2a* (serotonergic receptor), the *Grin1* gene (implicated in NMDA function), and different neuroplasticity markers such as *Bdnf*, *Ngf*, *Egr1*, *Arc*, and *Scn2a* in the FC. These changes point towards the fact that tDCS regulates neurotransmission in key circuits and promotes synaptic remodeling, in spite of not observing a significant reduction in compulsive behavior. The brain molecular changes promoted by tDCS could contribute to rewiring frontal circuits that might help in its therapeutic impact on compulsive spectrum disorders.

### Benefits and Limitations of the Study

Our study shows evidence of the underlying mechanisms of neurobehavioral effects induced by tDCS using preclinical models. Most preclinical studies using tDCS as a treatment usually place electrodes directly on the skull of the animals, although there are also some studies that choose to place the electrodes on the skin. This is intended to preserve ecological validity, equating with the procedures conducted on humans. However, in most of these studies, animals are anesthetized or immobilized using such cloths or tubes during the stimulation process [33, 63, 65, 107]. In contrast, it is relevant to note that one benefit of our study stands out for being the first in which stimulation is performed on the skin without applying any kind of restraint to the animal. The experimental animals have been habituated for tDCS application and allowed to remain free within an open space. This methodology is presented to ensure maximum ecological validity, as it more closely resembles clinical conditions.

However, our study presents some limitations. First, the reduction induced by tDCS treatment in compulsive drinking on SIP was insufficient; no significant differences were obtained when comparing the tDCS and sham groups of rats (*p* = 0.05). The result obtained in our study, a trend to reduce compulsive drinking on SIP, with a considerable number of animals used in each group (*n* = 15), points towards the need for future studies to consider including new assessments in other forms of compulsivity. Second, the tDCS procedure used in the present study was based on previous studies related to extinction behavior, highlighting the lack of work exploring the optimal timing of stimulation in preclinical models of compulsivity; future studies should investigate whether simultaneous treatment and behavioral assessment or different exposure schedules enhance therapeutic effects on compulsive behavior. Third, the absence of tDCS effects in the amygdala may reflect that we did not apply direct stimulation to this region and that our parameters may not have been sufficiently intense to induce plasticity in areas connected to the FC, such as the amygdala. Fourth, the small head size of the rat limited us to bimodal stimulation. Finally, we only assessed neuroplasticity and behavioral outcomes in male rats, not females. These findings underscore the need for future work to diversify behavioral paradigms, optimize stimulation timing and intensity, explore direct targeting of relevant brain regions, and include both sexes to fully characterize and translate tDCS effects in neuropsychiatric contexts.

## Conclusions

The present study revealed robust effects of anodal tDCS treatment on serotonin, glutamatergic, and neuroplasticity markers in the FC of compulsive rats. Anodal tDCS neurostimulation increased the expression levels of *Htr2a*, *Grin1*, *Bdnf*, *Ngf*, and *Scn2a* in the FC. However, tDCS treatment did not induce a significant reduction in water intake in compulsive rats on SIP. Considering that compulsivity is associated with a reduction in serotonin 5-HT2A receptor as well as other neuroplasticity biomarkers in the FC, the therapeutic effects of tDCS might be related to the neuroplasticity changes that might restore serotonin, glutamate, and neuroplasticity in the FC. Future studies should investigate whether tDCS can reduce compulsivity in other preclinical models and whether alternative stimulation tDCS protocols—considering simultaneous application during behavioral assessment or modifications in stimulation parameters—could lead to a significant reduction in compulsive drinking on the SIP paradigm. Moreover, future tDCS studies should examine the implication of the amygdala in the reduction of compulsivity—either through direct stimulation or indirectly via network-based modulation—and consider the individual differences in its activation as a potential factor that affects treatment efficacy. tDCS is a safe, cost-effective, and non-invasive technique capable of inducing neuroplastic adaptations that might translate into behavioral improvements without pharmacological intervention. However, further research is needed to determine how tDCS can be applied more effectively according to neuropsychiatric symptomatology.

## Data Availability

No datasets were generated or analysed during the current study.
